# Gene Expression Profiles Distinguish the Carcinogenic Effects of Aristolochic Acid in Target (Kidney) and Non-target (Liver) Tissues in Rats

**DOI:** 10.1186/1471-2105-7-S2-S20

**Published:** 2006-09-26

**Authors:** Tao Chen, Lei Guo, Lu Zhang, Leming Shi, Hong Fang, Yongming Sun, James C Fuscoe, Nan Mei

**Affiliations:** 1 Division of Genetic and Reproductive Toxicology, National Center for Toxicological Research, US FDA, Jefferson, AR 72079, USA; 2 Division of Systems Toxicology, National Center for Toxicological Research, US FDA, Jefferson, AR 72079, USA; 3 Molecular Biology SDS/Arrays Group, Applied BioSystems, Foster City, CA 94404, USA; 4 Z-Tech Corporation, 3900 NCTR Road, Jefferson, Arkansas 72079 USA; 5 Solexa, Inc., 25861 Industrial Boulevard Hayward, CA 94545, USA

## Abstract

**Background:**

Aristolochic acid (AA) is the active component of herbal drugs derived from *Aristolochia *species that have been used for medicinal purposes since antiquity. AA, however, induced nephropathy and urothelial cancer in people and malignant tumors in the kidney and urinary tract of rodents. Although AA is bioactivated in both kidney and liver, it only induces tumors in kidney. To evaluate whether microarray analysis can be used for distinguishing the tissue-specific carcinogenicity of AA, we examined gene expression profiles in kidney and liver of rats treated with carcinogenic doses of AA.

**Results:**

Microarray analysis was performed using the Rat Genome Survey Microarray and data analysis was carried out within ArrayTrack software. Principal components analysis and hierarchical cluster analysis of the expression profiles showed that samples were grouped together according to the tissues and treatments. The gene expression profiles were significantly altered by AA treatment in both kidney and liver (*p *< 0.01; fold change > 1.5). Functional analysis with Ingenuity Pathways Analysis showed that there were many more significantly altered genes involved in cancer-related pathways in kidney than in liver. Also, analysis with Gene Ontology for Functional Analysis (GOFFA) software indicated that the biological processes related to defense response, apoptosis and immune response were significantly altered by AA exposure in kidney, but not in liver.

**Conclusion:**

Our results suggest that microarray analysis is a useful tool for detecting AA exposure; that analysis of the gene expression profiles can define the differential responses to toxicity and carcinogenicity of AA from kidney and liver; and that significant alteration of genes associated with defense response, apoptosis and immune response in kidney, but not in liver, may be responsible for the tissue-specific toxicity and carcinogenicity of AA.

## Background

Aristolochic acid (AA) is an active component of herbal drugs derived from *Aristolochia *species that have been used for medicinal purposes since antiquity. The herbal drugs containing AA were used for treatment of snake bites, arthritis, gout, rheumatism and festering wounds, as well as used in obstetrics [1]. This compound, however, is a nephrotoxin and carcinogen. A progressive form of renal fibrosis is associated with patients taking weight-reducing pills containing AA [2-4]. The aristolochic acid nephropathy was initially observed in Belgium in 1991 and about half of the patients had renal replacement therapy [2,5,6]. Later, this disease also was found in other European countries, and in Asia and the USA [1]. A high prevalence of urothelial carcinoma was found in aristolochic acid nephropathy patients [3,7,8]. Animal models also demonstrated that AA resulted in renal failure in rodents [9], and tumors in the kidney, forestomach, and other tissues of rats and mice [10-12]. AA was identified among the most potent 2% of carcinogens [13]. The International Agency for Research on Cancer (IARC) has classified the products containing AA as human carcinogens [14].

Due to the toxicity of AA, the Food and Drug Administration (FDA) advised consumers to immediately discontinue use of any botanical products containing AA and published a list of botanical products that contained AA in 2001 [15]. Despite the FDA's warning, many products containing or suspected to contain AA can still be identified on U.S. Web sites for sale for gastrointestinal symptoms, weight loss, cough, and immune stimulation [16].

AA is bioactivated and subsequently reacts with cellular proteins and DNA, leading to multiple forms of toxicity. AA is activated and produces DNA adducts in both kidney and liver [17-20]. It, however, induces tumor only in kidney although liver is the major organ for biotransformation of xenobiotics. The underlying mechanisms for the tissue-specific carcinogenicity of AA are unknown.

The advent of gene microarrays permits the analysis of gene expression patterns for thousands of genes simultaneously in biological samples of interest, providing new insights into the effects of chemicals on biological systems and allowing the macrodissection of molecular events in chemical carcinogenesis. Identification of unique gene expression patterns produced by carcinogens may allow us to elucidate mechanisms of action. In this study, we treated rats with a carcinogenic dose of AA and conducted microarray analysis of gene expression in the kidney and liver. To explore whether gene expression profiles can be used for identifying AA exposure and to elucidate the tissue-specific carcinogenicity of AA, clustering analysis, functional analysis and biological process analysis were performed on the gene expression profiles of kidney and liver of rats treated with AA and the vehicle control. We found that the gene expression profiles were significantly altered by AA treatment in both kidney and liver, and many more significant genes involved in cancer-related pathways were found in kidney than in liver. Analysis of biological process reveals that genes that are related to apoptosis and immune response are largely altered by AA treatment in kidney, but not in liver.

## Results and Discussion

To investigate the effect of AA exposure on gene expression in the target tissue and non-target tissue, we treated rats with a protocol similar to one that resulted in tumors in rats [11]. Six-week-old rats were treated with 10 mg AA/kg body weight five days a week for 12 weeks. One day after the last treatment, 6 animals from the treated group and 6 animals from the vehicle control group were sacrificed, and the kidney (target tissue) and liver (non-target tissue) were removed for the microarray analysis. In the original carcinogenesis study [11], atypical cells and hyperplasia were found in the kidney immediately after cessation of AA treatment while adenomas and adenocarcinomas were not observed until three months after the cessation of AA treatment. Because the sampling time in the present study was one day after the three month treatment, it is expected that many different stages of tumor formation induced by AA will be present. The alteration of gene expression profiles should reflect this process in kidney and liver.

### Principal component analysis and hierarchical cluster analysis group the samples according to tissues and AA treatment

**Figure 1:**
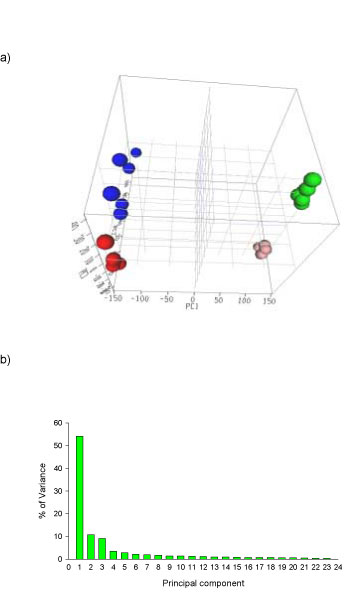
Principal component analysis (PCA) for gene expression profiles from kidney and liver of rats treated with aristolochic acid and their concurrent controls. a) 3D view. The autoscaled method was used for the PCA. The red, blue, pink and green dots indicate kidney control, kidney with aristolochic acid (AA) treatment, liver control, and liver with AA treatment, respectively. PC1, PC2, and PC3 represent first principal component, second principal component, and third principal component, respectively. b) Distribution of variance in principal components.

**Figure 2:**
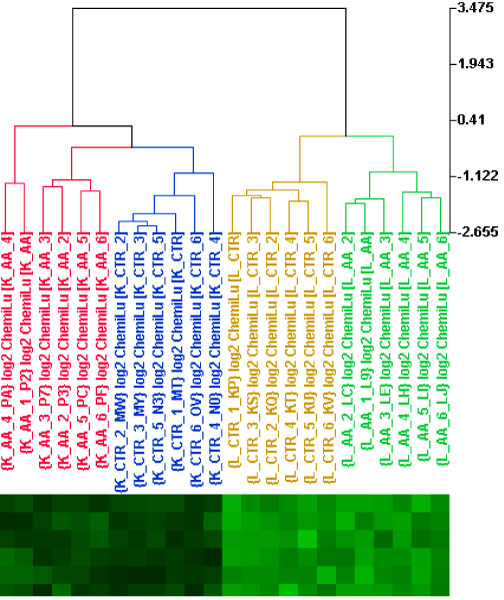
Hierarchical clustering of samples according to gene expression profiles from kidney and liver of rats treated with aristolochic acid and their concurrent controls. Ward's Minimum Variance method was used. Labels K_AA, K_CTR, L_AA, and L_CTR indicate kidney with AA-treatment, kidney control, liver with AA-treatment and liver control, respectively.

Principal component analysis (PCA) was used to visualize clusters of samples corresponding to the tissues and treatment of AA based on variance-covariance structure of the gene expressions in the 24 samples. PCA uses analysis of the principal sources of variance in data and displays this information graphically either 2-dimensionally or 3-dimensionally [21]. A PCA 3D view using the first three principal components for gene expression profiles from the samples is displayed in Figure 1a. The PCA is based on log2 intensity and the expression profiles are across 14361 genes whose signal intensities were greater than 150. The captured variances of the first three components reached 74% of the total variance (Figure 1b), indicating the three components were able to represent most of the expression pattern of the individual samples. A hierarchical cluster analysis (HCA) within ArrayTrack also was also used to cluster the samples (Figure 2).

The PCA and HCA results demonstrate that samples were grouped together according to the tissues and AA treatment. The kidney samples were well separated from the liver samples, suggesting a large difference between expression patterns of the two tissues. AA treatment resulted in a clear alteration of gene expression in both kidney and liver (Figures 1a and 2). These data suggest that AA exposure in kidney and liver can be identified based on gene expression profiles.

### Genes associated with carcinogenesis were differentially regulated by AA treatment in kidney and liver

While the PCA and HCA show that AA treatment altered the gene expression patterns, it does not reveal how many genes and what kinds of genes are modulated and what the differences are between kidney and liver. To this end, the expression levels of genes in kidney and liver of rats treated with AA were evaluated.

**Figure 3:**
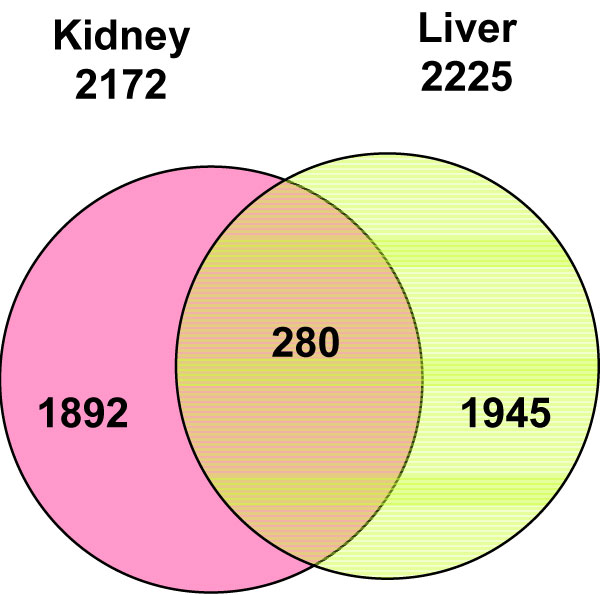
Venn diagram for genes whose expression was significantly altered by aristolochic acid in kidney and liver. Gray, pink and yellow colors indicate genes whose expressions were altered in both kidney and liver, only kidney and only liver, respectively.

The differentially expressed genes between the treatment and control (significant genes) were identified based on a cutoff of *p *< 0.01 and fold change > 1.5. Among the 26,857 genes, a total of 2172 and 2225 significant genes were found, with 1063 and 914 genes down-regulated and 1109 and 1311 genes up-regulated in kidney and liver, respectively. When the kidney gene expression profile was compared to the liver profile, 280 genes were found to be commonly altered in both kidney and liver, with most (204 genes) increasing or decreasing concordantly in the both tissues (Figure 3).

The different sets of genes that were significantly altered by AA in kidney and liver were likely the results of tissue-specific response to AA treatment and the different tissue-specific suite of active genes present in those tissues. For example, gene expression was very different between the kidney and liver of control rats, with about 38% of genes being differentially expressed (*p *< 0.01; fold change >1.5). PCA and HCA results have also demonstrated the large difference between expression patterns of the two tissues (Figures 1 and 2). It would be expected that AA treatment would impact the expression of some genes that are uniquely expressed in the two tissues. Also, tissue-specific response to AA exposure would produce different significant genes. Rat kidney has been identified as a target tissue for AA carcinogenesis while liver is a major site for AA metabolism. It would be expected that more cancer-related genes were altered by AA treatment in kidney than in liver.

To compare the extent of the gene expression changes caused by AA in kidney and liver, the significant genes were grouped by fold-change (Table 1). There were more significant genes with higher fold-changes in kidney than in liver, indicating that AA treatment of the rats resulted in greater changes in gene expression in kidney than in liver. This difference might play an important role in the differential carcinogenicity and toxicity of AA in kidney and liver.

The large number of significant genes resulting from AA treatment in kidney and liver are expected to be the result of different properties of AA, including pharmacological and toxicological effects of this chemical. To identify the genes related to carcinogenic effects of AA, we analyzed the significant genes with Ingenuity Pathways Analysis software (IPA, Ingenuity Systems, Redwood City, CA). Functional analysis with IPA showed that there were many more significant genes involved in cancer-related pathways in kidney than in liver (Table 2). A total of 372 significant genes in kidney and 111 significant genes in liver were involved in functions associated with different stages of carcinogenesis. These functions included AA metabolism, DNA repair, cell cycle, cellular development, cell signaling, cellular growth and proliferation, cell morphology, cell death, and tumor morphology.

Chemical carcinogenesis is a multistage process, with initiation, promotion and progression. Certain genes can be involved in a specific carcinogenic process and their expression changes can be indicative of the process. Due to our chronic treatment schedule, many of the different stages of AA carcinogenesis are expected to be detected at our sampling time. The different number of genes associated with carcinogenesis between kidney and liver correlates with the differential carcinogenicity of AA in the two tissues.

### Biological process analysis revealed that the defense response processes was significantly changed by AA exposure in kidney, but not in liver

Gene Ontology for Functional Analysis (GOFFA) within ArrayTrack was developed at the National Center for Toxicological Research (NCTR), Jefferson, AR. This software orders GO (gene ontology) terms by prevalence for a list of selected genes or proteins, and then allows the user to interactively select GO terms according to their significance and specified biological complexity within the hierarchical structure. GO has established a vocabulary that provides a hierarchical structure for the analysis of genome data and provides a classification of gene products into molecular functions, biological processes, and cellular components [22] although GO curation of known literature is incomplete and limited to gene function/localization/process. GOFFA uses the GO database for obtaining overviews of data and providing a rapid mechanism for researchers to classify genes that are often given non-descriptive numerical names during genome annotation. Significant gene lists were generated using criteria of *p *< 0.01 and fold change > 2.0. These lists were directly utilized for GOFFA analysis. Biological process in GOFFA terms was examined for the genes from kidney and liver individually. Terms that are significantly altered (*p *< 0.01) are summarized in Table 3 and Table 4.

**Figure 4:**
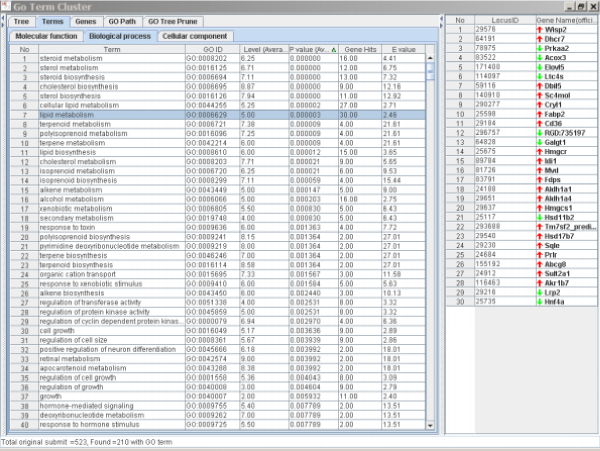
Significantly altered terms and the genes associated with lipid metabolism caused by AA treatment in rat liver. The left panel is the Terms Window and the right panel is the Genes Window. The major genes in the list were up-regulated.

**Figure 5:**
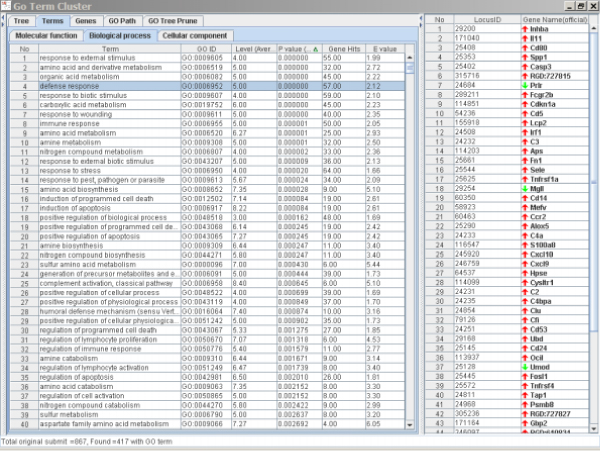
Significantly altered terms and the genes associated with defense response caused by AA treatment in rat kidney. The left panel is the Terms Window and the right panel is the Genes Window. The major genes in the list were up-regulated.

**Figure 6:**
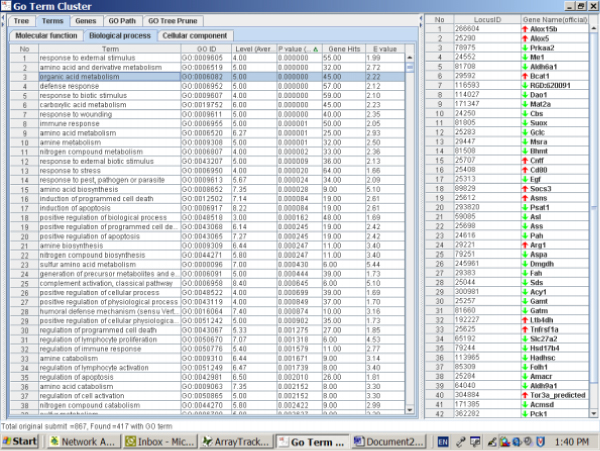
Significantly altered terms and the genes associated with organic acid metabolism caused by AA treatment in rat kidney. The left panel is the Terms Window and the right panel is the Genes Window. The major genes in the list were down-regulated.

By comparing the alterations in biological processes in the kidney and liver associated with AA exposure (Table 3 and Table 4), it can be seen that the changes were very different for these two tissues. These changes appear to be related to different effects of AA on each tissue and the tissue-specific responses to AA. Most of the altered biological processes in liver were related to lipid metabolism including steroid and other lipid metabolisms. Most of these genes were up-regulated (Figure 4), which might result from the pharmacological effects of AA because AA is an inhibitor of phospholipase A 2 proteins that can hydrolyze phospholipids to form fatty acid and lysophospholipid products. The most significantly changed processes associated with AA exposure in kidney, however, were defense response (including apoptosis and immune responses, etc.) and organic acid metabolism (amino acid and carboxylic acid metabolisms, etc.). The genes in the pathways for response to stress, toxin, biotic stimulus, tumor induction, and immune response were mainly up-regulated by AA treatment (Figure 5) whereas the genes in the organic acid metabolism were mainly down-regulated (Figure 6).

It is not unexpected that AA treatment triggers a strong defense response in kidney considering that AA is a potent nephrotoxin and kidney carcinogen [1,2,5,7]. Alteration of immune response is a common biological reaction to tissue damage, toxicity and tumors, while apoptosis is a biological process that responds to toxicity, especially for DNA damage. A large number of genes involved in these processes can indicate the carcinogenic insults. AA-induced apoptosis activities in cell culture and in tubular cells of kidney have been reported [23-25] and tubular cell apoptosis has been considered one of the mechanisms for AA renal injury [26]. It has also been reported that AA can induce mutations in the *p*53 and H-*ras *genes [1] that are related to alteration of apoptosis [27-29]. The genes involved in apoptosis were generally up-regulated to remove the damaged cells caused by AA treatment. For example, the *inhba *gene (first gene in the right panel of Figure 5) whose expression increased 4.1-fold over the control by AA treatment in kidney is a tumor-suppressor gene and it produces a protein that increases growth arrest in tumor cells [30]. Therefore, its induction in the kidney by AA exposure may indicate a tissue response to genotoxic damage.

The reasons for the down-regulation of genes for organic acid metabolism in kidney due to AA exposure are unknown. A recent metabonomic study using urine and blood samples from rats treated with AA [31] indicates that certain metabolic pathways involving organic acids, such as homocysteine formation and the folate cycle, were significantly accelerated while others, including arachidonic acid biosynthesis, were decreased. These alteration has been associated with AA-induced nephrotoxicity [31]. Therefore, a number of genes involving amino acid and carboxylic acid metabolisms might also be indicative of AA-induced nephrotoxicity.

## Conclusion

We analyzed gene expression profiles in kidney and liver tissues of rats treated with AA and vehicle control by the systematic approaches of PCA, functional analysis, and biological process analyses. Our findings demonstrate that AA treatment produced a significant alteration of gene expression in both kidney and liver. The changes of gene expressions induced in kidney and liver of rats, however, were different, which may indicate tissue-specific mechanisms of AA carcinogenicity. There were many more significant genes associated with carcinogenesis in kidney than in liver due to AA exposure. Significant changes in biological processes related to defense response, apoptosis and immune response, as well as organic acid metabolism were found in kidney, but not in liver. These differential alterations between kidney and liver could be the underlying mechanisms for the tissue-specific toxicity and carcinogenicity of AA.

## Materials and methods

### Animal and treatment

Big Blue transgenic Fisher 344 rats were obtained from Taconic Laboratories (Germantown, NY). The transgenic rats were chosen because the same animals were also utilized for a study on mutagenicity of AA. We followed the recommendations of the NCTR Institutional Animal Care and Use Committee for the handling, maintenance, treatment, and sacrifice of the rats.

The treatment schedule was based on the previous carcinogenicity study [11]. Six 6-week-old male Big Blue rats were treated with AA (Sigma, St. Louis, MO) as its sodium salt at concentrations of 10 mg/kg body weight by gavage (4 ml/kg body weight) five times a week for 12 weeks. Six control rats were gavaged with the vehicle, 0.9% sodium chloride, using the same schedule. The rats were sacrificed one day after the last treatment; the kidneys and livers were isolated, frozen quickly in liquid nitrogen, and stored at -80°C.

### RNA isolation and quality control

Total RNA from liver and kidney of the treated and control rats was isolated using an RNeasy system (Qiagen, Valencia, CA). The yield of the extracted RNA was determined spectrophotometrically by measuring the optical density at 260 nm. The quality of the extracted RNA was evaluated using the RNA 6000 LabChip and Agilent 2100 Bioanalyzer (Agilent Technologies, Palo Alto, CA). Only RNA with RNA integrity numbers (RINs) greater than 7.5 were used for microarray experiments. The microarray analysis was performed using Applied Biosystems' Rat Genome Survey Microarray with 26,857 gene probes.

### Preparation of digoxigenin labeled cRNA

All RNA targets were labeled using the Applied Biosystems RT-IVT Labeling Kit Version 2.0. Briefly, 1.5 μg of total RNA sample was reverse transcribed by 2 h incubation at 42°C with ArrayScript RT™ enzyme (Ambion, Austin, TX) and oligo dT-T7 primer. Double-stranded cDNA was produced following 2 h incubation with *E. coli *DNA polymerase and RNase H at 16°C. The double-stranded cDNA was purified using RT-IVT kit following the manufacturer's protocol. The in vitro transcription was performed by incubation of the cDNA product with T7 RNA polymerase, 0.75 mM Digoxigenin-11-UTP (Roche Applied Science, Indianapolis, IN) and all other NTPs for 9 h. Labeled cRNA was purified according to the RT-IVT kit protocol and analyzed for quality and quantity using standard UV spectrometry and the Bioanalyzer.

### Hybridization of labeled cRNA to microarrays and microarray imaging

Digoxigenin labeled cRNA targets were hybridized to Applied Biosystems Rat Whole Genome Survey Microarrays and detected using the Applied Biosystems Chemiluminescent Detection Kit. Briefly, 15 μg of labeled cRNA targets were fragmented via incubation with fragmentation buffer provided in the kit for 30 min at 60°C. Fragmented targets were hybridized to microarrays during 16 h incubation at 55°C with buffers and reagents from the Chemiluminescent Detection Kit. Post-hybridization washes and anti-Digoxigenin-Alkaline Phosphatase binding were performed according to the protocol of the kit. Chemiluminescence detection, image acquisition and analysis were performed using Applied Biosystems Chemiluminescence Detection Kit and Applied Biosystems 1700 Chemiluminescent Microarray Analyzer following the manufacturer's protocols. Images were auto-gridded and the chemiluminescent signals were quantified, corrected for background, and finally, spot- and spatially-normalized using the Applied Biosystems 1700 Chemiluminescent Microarray Analyzer software version 1.1.

### Normalization and statistic analysis

Gene expression data from the Applied Biosystems' Rat Genome Survey Microarray were input to ArrayTrack for the management, analysis, visualization and interpretation of microarray data [32]. Raw microarray intensity data were normalized per chip to the same median intensity value of 500. The identification of differentially expressed genes was based on permutation *t*-test. We evaluated several statistical methods to determine the significant genes including Welch *t*-test, permutation *t*-test and SAM (Significance Analysis of Microarrays [33]), all of which yielded a set of similar significant genes. Since the genes generated from permutation *t*-test covered 96% of significant genes from Welch *t*-test and 97% significant genes from SAM, permutation *t*-test was chosen for this study. For the permutation tests, fullpermutations of 12 samples (6 for treatment and 6 for control) were performed on the dataset of each tissue. The significant genes were selected using cutoffs of *p *< 0.01 and fold change > 1.5.

### Principal component analysis (PCA) and hierarchical cluster analysis (HCA)

PCA were conducted using the autoscaled method within ArrayTrack [34]. The normalized data were converted into log2 ratios and the data was filtered with signal intensity. About 14361 genes (channel intensities > 150) in the arrays were used for the PCA and HCA.

### Functional analysis of the significant genes with IPA

Functional analysis of significant genes was performed with IPA, a web-delivered software for discovering, visualizing, and exploring relevant functions, pathways and networks [35]. Significant genes were uploaded into IPA. LocusID of each gene was mapped to its corresponding gene object in the IPA Knowledge Base. A total of 911 out of 2172 and 845 out of 2225 significant genes in kidney and liver, respectively, were eligible for IPA analysis. These genes were then used as the starting point for generating biological functions, pathways and networks. Biological functions were calculated and assigned to different networks. Genes related to carcinogenesis were identified and used for comparing the carcinogenic processes in kidney and liver of rats treated with AA.

### Analysis of Biological process with GOFFA

GOFFA software within ArrayTrack provides gene ontology information using the standard vocabulary (terminology) of the Gene Ontology Consortium. The ontology provides standard vocabularies for the description of the molecular function, biological process and cellular component of gene products. ArrayTrack is freely available at . Detailed descriptions of the GOFFA tool, including the statistical analysis methods, are available in the user's manual at the above web site. Lists of genes whose expression was significantly altered in the kidney or liver by AA exposure were generated using a gene selection cutoff of *p *< 0.01 and fold change > 2.0, GOFFA was then used to analyze the biological processes affected by AA treatment in liver and kidney. Significant biological processes were selected from GOFFA Terms (*p *< 0.01) for distinguishing differential response of kidney and liver to AA exposure.

## Authors' contributions

TC performed the analysis of microarray data and wrote the manuscript. LG, LS, HF, and JCF helped conceive the experiments, analyze the data and write the manuscript. LZ, YS conducted the microarray experiment and generated the raw data. NM performed the animal treatment and isolation of tissues and total RNA, and was involved in designing the experiment and writing the manuscript. All authors read and approved the final version of manuscript.

## Figures and Tables

**Table 1:** Fold-changes of genes whose expression levels were altered significantly by aristolochic acid in kidney and liver.

Fold change	Number of genes	
	
	Kidney	Liver
> 10	29	16
5–10	95	34
2–5	1050	788
1.5–2	998	1387
Total	2172	2225

**Table 2:** Numbers of genes whose functions are related to carcinogenesis and whose expression was altered by aristolochic acid in kidney and liver of rats.

***Functions***	***Kidney***	***Liver***
Drug Metabolism	14	10
DNA replication, recombination and repair	2	4
Genetic disorder	17	6
Cell cycle	26	13
Cellular development	18	7
Cellular function and maintenance	16	5
Cell-to-cell and signaling and interaction	41	8
Cell morphology	36	15
Cell death	54	2
Cellular growth and proliferation	37	8
Tumor morphology	19	8
Cancer	92	25
Total	372	111

Note: the same genes may exist in different functions.

**Table 3:** List of significant biological processes generated with GOFFA Terms in kidney of rats treated with aristolochic acid (*p *< 0.01).

Go term	GO ID	P value (Average)	None of Genes Involved
Response to external stimulus	GO:0009605	0.000000	55
Amino acid and derivative metabolism	GO:0006519	0.000000	32
Organic acid metabolism	GO:0006082	0.000000	45
Defense response	GO:0006952	0.000000	57
Response to biotic stimulus	GO:0009607	0.000000	59
Carboxylic acid metabolism	GO:0019752	0.000000	45
Response to wounding	GO:0009611	0.000000	40
Immune response	GO:0006955	0.000001	50
Amino acid metabolism	GO:0006520	0.000001	25
Amine metabolism	GO:0009308	0.000001	32
Nitrogen compound metabolism	GO:0006807	0.000002	33
Response to external biotic stimulus	GO:0043207	0.000009	36
Response to stress	GO:0006950	0.00002	64
Response to pest, pathogen or parasite	GO:0009613	0.000024	34
Amino acid biosynthesis	GO:0008652	0.000028	9
Induction of programmed cell death	GO:0012502	0.000084	19
Induction of apoptosis	GO:0006917	0.000084	19
Positive regulation of biological process	GO:0048518	0.000162	48
Positive regulation of programmed cell death	GO:0043068	0.000245	19
Positive regulation of apoptosis	GO:0043065	0.000245	19
Amine biosynthesis	GO:0009309	0.000247	11
Nitrogen compound biosynthesis	GO:0044271	0.000247	11
Sulfur amino acid metabolism	GO:0000096	0.000430	6
Generation of precursor metabolites and energy	GO:0006091	0.000444	39
Complement activation, classical pathway	GO:0006958	0.000645	6
Positive regulation of cellular process	GO:0048522	0.000699	39
Positive regulation of physiological process	GO:0043119	0.000849	37
Humoral defense mechanism (sensu Vertebrata)	GO:0016064	0.000874	10
Positive regulation of cellular physiological process	GO:0051242	0.000902	35
Regulation of programmed cell death	GO:0043067	0.001275	27
Regulation of lymphocyte proliferation	GO:0050670	0.001318	6
Regulation of immune response	GO:0050776	0.001579	11
Amine catabolism	GO:0009310	0.001671	9
Regulation of lymphocyte activation	GO:0051249	0.001739	8
Regulation of apoptosis	GO:0042981	0.002010	26
Amino acid catabolism	GO:0009063	0.002152	8
Regulation of cell activation	GO:0050865	0.002152	8
Nitrogen compound catabolism	GO:0044270	0.002422	9
Sulfur metabolism	GO:0006790	0.002637	8
Aspartate family amino acid metabolism	GO:0009066	0.002692	4
Negative regulation of biological process	GO:0048519	0.002726	45
Electron transport	GO:0006118	0.002754	23
Cellular catabolism	GO:0044248	0.002821	27
Xenobiotic metabolism	GO:0006805	0.003190	6

**Table 4:** List of significant biological processes generated with GOFFA Terms in liver of rats treated with aristolochic acid (*p *< 0.01).

Go term	GO ID	P value (Average)	Number of Genes Involved
Steroid metabolism	GO:0008202	0.000000	16
Sterol metabolism	GO:0016125	0.000000	12
Steroid biosynthesis	GO:0006694	0.000000	13
Cholesterol biosynthesis	GO:0006695	0.000000	9
Sterol biosynthesis	GO:0016126	0.000000	11
Cellular lipid metabolism	GO:0044255	0.000002	27
Lipid metabolism	GO:0006629	0.000003	30
Terpenoid metabolism	GO:0006721	0.000009	4
Polyisoprenoid metabolism	GO:0016096	0.000009	4
Terpene metabolism	GO:0042214	0.000009	4
Lipid biosynthesis	GO:0008610	0.000012	15
Cholesterol metabolism	GO:0008203	0.000021	9
Isoprenoid metabolism	GO:0006720	0.000021	6
Isoprenoid biosynthesis	GO:0008299	0.000059	4
Alkene metabolism	GO:0043449	0.000147	5
Alcohol metabolism	GO:0006066	0.000203	16
Xenobiotic metabolism	GO:0006805	0.000830	5
Secondary metabolism	GO:0019748	0.000830	5
Response to toxin	GO:0009636	0.001363	4
Polyisoprenoid biosynthesis	GO:0009241	0.001364	2
Pyrimidine deoxyribonucleotide metabolism	GO:0009219	0.001364	2
Terpene biosynthesis	GO:0046246	0.001364	2
Terpenoid biosynthesis	GO:0016114	0.001364	2
Organic cation transport	GO:0015695	0.001567	3
Response to xenobiotic stimulus	GO:0009410	0.001584	5
Alkene biosynthesis	GO:0043450	0.002440	3
Regulation of transferase activity	GO:0051338	0.002531	8
Regulation of protein kinase activity	GO:0045859	0.002531	8
Regulation of cyclin dependent protein kinase activity	GO:0000079	0.002970	4
Cell growth	GO:0016049	0.003636	9
Regulation of cell size	GO:0008361	0.003939	9
Positive regulation of neuron differentiation	GO:0045666	0.003992	2
Retinal metabolism	GO:0042574	0.003992	2
Apocarotenoid metabolism	GO:0043288	0.003992	2
Regulation of cell growth	GO:0001558	0.004043	8
Regulation of growth	GO:0040008	0.004604	9
Growth	GO:0040007	0.005932	11
Hormone-mediated signaling	GO:0009755	0.007789	2
Deoxyribonucleotide metabolism	GO:0009262	0.007789	2
Response to hormone stimulus	GO:0009725	0.007789	2
G2/M transition of mitotic cell cycle	GO:0000086	0.007897	4
Electron transport	GO:0006118	0.009494	13
